# The efficacy of extracorporeal shockwave lithotripsy for symptomatic ureteral stones: Predictors of treatment failure without the assistance of computed tomography

**DOI:** 10.1371/journal.pone.0184855

**Published:** 2017-09-20

**Authors:** Bing-Juin Chiang, Chun-Hou Liao, Yu-Hua Lin

**Affiliations:** 1 Department of Life Science, National Taiwan Normal University, Taipei, Taiwan; 2 Division of Urology, Department of Surgery, Cardinal Tien Hospital, New Taipei City, Taiwan; 3 Department of Urology, School of Medicine, Fu-Jen Catholic University, New Taipei City, Taiwan; 4 Department of Urology, National Taiwan University Hospital, Taipei, Taiwan; Leibniz-Institut fur Pflanzengenetik und Kulturpflanzenforschung Gatersleben, GERMANY

## Abstract

**Objectives:**

Non-contrast computed tomography (NCCT) is not always performed clinically if the diagnosis of ureteral calculi has been confirmed using other radiographic imaging modalities. The aim of this study was to identify predictors of successful extracorporeal shockwave lithotripsy (ESWL) without assistance of NCCT.

**Methods:**

We retrospectively reviewed the medical records of patients with symptomatic solitary ureteral stones who underwent ESWL between November 2015 and January 2016. Abdominal plain radiography or intravenous urography were performed before ESWL for localization. The exclusion criteria were repeated sessions of ESWL for the target stone and congenital genitourinary tract anomalies. The demographic characteristics, clinical history, medical charges, or imaging features of the stones were recorded. Successful treatment was defined as no fragments detected on radiography or ultrasonography in 4 weeks. For radiolucent calculi, successful treatment was regarded as cases without hydronephrosis, symptoms, or hematuria. Patients experiencing intractable pain and undergoing subsequent auxiliary surgeries were regarded as having ESWL treatment failure.

**Results:**

Age (odds ratio [OR], 1.042; 95% confidence interval [CI], 1.007–1.078), history of ipsilateral renal or ureteral calculi episodes (OR, 2.669; 95% CI, 1.281–5.687), stone burden (OR, 3.499; 95% CI, 1.284–9.530), and radiopaque stone (OR, 2.351; 95% CI, 1.049–5.267) were significant predictors of ESWL failure in all patients.

**Conclusions:**

For treating symptomatic ureteral stones, those with smaller size, radiolucency, and without a history of ipsilateral renal or ureteral calculi could be considered for first-line therapy with ESWL.

## Introduction

Non-contrast computed tomography (NCCT) is believed to be a more effective tool than intravenous urography (IVU) in the evaluation of acute renal colic[1]. It has become a popular investigation for emergency department (ED) patients presenting with suspected renal colic. It also has been accepted as the standard imaging investigation of choice by the Royal College of Radiologists (RCR) iRefer guidelines in the UK, and the American College of Radiologists Appropriateness Criteria [2]. Recent research has investigated possible predictors for successful extracorporeal shockwave lithotripsy (ESWL) treatment, including the consistency, size, shape, location, and density in Hounsfield units (HU) of the ureteral stone; and skin-to-stone distance (SSD), which were evaluated by NCCT in more than half of all published studies [3–5]. With a sensitivity of 94–100% and specificity of 92–100%, NCCT is more effective than IVU as a first-line confirmation tool for ureteral stones in the emergency department[1]. However, IVU is more convenient than NCCT as an intra-operative adjunct for radiolucent ureteral stones if ultrasonic localization is not possible[6]. NCCT may be less accurate in localizing the suspected calculi due to impaired resolution of soft tissue structures [7].Radiation exposure is also around 1.5 times higher for NCCT than for IVU [8]. The detection of calculi was significantly lower (18 to 31%) in females investigated with NCCT for suspected renal colic [9]. NCCT is futile as an adjunctive intraoperative imaging modality, and is not always performed in current practice if the diagnosis has already been confirmed with plain radiography and ultrasonography. In Taiwan, NCCT is unnecessary prior to ESWL for National Health Insurance medical claims reviews. Thus, there is a difference between the accumulated evidence of predictors based on NCCT and those available in clinical practice.

For small ureteral calculi, extracorporeal shock wave lithotripsy (ESWL) has a comparable efficacy to ureteroscopic lithotripsy (URSL) [10]. Rapid ESWL within 24 hours in renal colic patients increases treatment success, reducing the risk of edema developing[11]. The advantages of ESWL include its noninvasiveness, low technical threshold, and no requirement for anesthesia. However, the lack of consistency in success rates may be related to the differing characteristics of calculi, patients, and performance of ESWL. Retreatment following unsuccessful ESWL treatment increases costs and affects quality of life. Therefore, predictors for successful ESWL treatment have an important role in decision making for patients with ureteral stones. We prospectively collected easily accessible parameters including history of ipsilateral renal or ureteral calculi episodes, weight, height, body mass index (BMI), waist circumference (WC), and buttock circumference (BC), in addition to the general characteristics of patients and the properties of calculi on radiography since November 2015 before ESWL.

## Materials and methods

### Study protocol

We retrospectively reviewed the medical records of patients undergoing ESWL between November 2015 and January 2016 in Cardinal Tien Hospital, New Taipei City. All patient records were de-identified and analyzed anonymously. The inclusion criteria were as follows: (1) a symptomatic solitary ureteral stone measuring 4 to 20 mm, (2) radiopaque calculi located within the ureter on abdominal plain radiography identified within one month before ESWL, or radiolucent calculi identified and measured on IVU immediately before ESWL. Patients who had undergone repeated sessions of ESWL for the target stone, those with congenital genitourinary tract anomalies, and those who were pregnant were excluded. Patient characteristics, including age, gender, BMI, WC, BC, history of ipsilateral renal or ureteral calculi episodes, and history of underlying systemic disease, were collected. WC was measured as a horizontal line at a level midway between the lowest rib and the iliac crest. BC was measured at the level of the greater trochanters. Variables including stone location, stone length, stone width, and maximal energy delivered were recorded. Stone length was defined as the longest diameter measured on radiography. Stone width was measured as the diameter perpendicular to the length. Stone burden was calculated by multiplying the length by the width. The medical billing statements were also reviewed after all medical charges were compiled. The costs were based on ESWL, radiographs, office visits, and other required medicines during the periods. The study protocol was approved by the Research Ethics Committee of Cardinal Tien Hospital.

### Treatment

All ESWL treatments were performed using the Dornier Compact Delta-lithotripter without anesthesia in outpatient clinic settings. Patients were treated with up to 3,000 shocks with a gradual power increase to a maximum of 13 kV at 90 shocks per minute if tolerated. The maximal energy applied in the ESWL session was recorded. We used IVU for localization of radiolucent calculi after a bolus injection of 1 ml/kg of contrast medium. If radiolucent calculi were identified on IVU, they were targeted with bi-planar fluoroscopy during ESWL. Tamsulosin hydrochloride (0.2 mg) and furosemide (40 mg) were prescribed for 7 days as routine medications. Clinic appointments for radiography, ultrasonography, and urine analysis surveillance were arranged for 4 weeks ± 1 week after ESWL. Successful treatment was defined as no fragments detected on radiography or ultrasonography. For radiolucent calculi, successful treatment was regarded as cases without hydronephrosis, symptoms, or hematuria. Patients experiencing intractable pain and undergoing subsequent auxiliary surgeries were regarded as having ESWL treatment failure.

### Statistical analysis

Results were analyzed with commercial statistical software (SPSS version 22.0 for Windows, SPSS, Chicago, IL, USA), and p-values were calculated with Chi-square analysis and Student t-test for the univariate analysis between successful treatments and all other cases. Factors found to be significant or potentially significant (p < 0.1), were further analyzed with multivariate regression analysis. Two-sided tests were used and a statistically significant difference was defined as p < 0.05.

## Results

From November 2015 to January 2016, 180 consecutive patients with a symptomatic solitary ureteral calculus underwent ESWL in this study. Six patients who failed to attend follow-up were excluded, leaving 174 patients who were enrolled. The demographic data are shown in Table 1. The mean stone length and width were 0.74 ± 0.35 and 0.42 ± 0.23 cm, respectively. The mean medical cost of the treatment procedure in the study was 32413 New Taiwan Dollars (NTDs), ranges from 27310 to 35538 NTDs.

**Table 1:** Demographics of all the patients, successful and failure treatment groups.

	Ureter stone n = 174	Success n = 114	Fail n = 60	P value
Patient characteristics				
Age (year)	49.1 ± 11.0	47.3 ± 10.9	52.5 ± 10.7	0.003
Male gender (%)	124 (71.3%)	84 (73.7%)	40 (66.6%)	0.379
Height (cm)	167.0 ± 7.9	167.5 ± 7.9	165.9 ± 7.8	0.199
Weight (kg)	74.2 ± 12.8	74.4 ± 13.2	73.7 ± 12.2	0.720
Waist circumference (cm)	89.3 ± 10.0	88.5 ± 9.8	90.8 ± 10.2	0.154
Buttock circumference (cm)	96.8 ± 7.9	96.2 ± 8.2	97.9 ± 7.3	0.179
BMI	26.5 ± 3.8	26.5 ± 3.9	26.7 ± 3.7	0.702
Recurrence^a^	91 (52.3%)	50 (43.9%)	41 (68.3%)	0.002
Diabetes	15 (8.6%)	7 (6.1%)	8 (13.3%)	0.154
Hypertension	36 (20.7%)	20 (17.5%)	16 (26.7%)	0.172
Calculi characteristics				
Length (cm)	0.74 ± 0.35	0.64 ± 0.25	0.91 ± 0.44	< 0.001
Width (cm)	0.42 ± 0.23	0.36 ± 0.22	0.54 ± 0.22	< 0.001
Stone burden (cm^2^)	0.38 ± 0.42	0.28 ± 0.35	0.56 ± 0.47	< 0.001
Radiolucent calculi (%)	65 (37.4%)	52 (45.6%)	13 (21.7%)	0.002
Position				
Right side (%)	81 (46.6%)	55 (48.2%)	26 (43.3%)	0.632
Proximal ureter (%)	79 (45.4%)	48 (42.1%)	31 (51.7%)	0.263
Middle ureter (%)	16 (9.2%)	8 (7.0%)	8 (13.3%)	0.179
Distal ureter (%)	79 (45.4%)	58 (50.8%)	21 (35.0%)	0.055
Maximal energy applied (kv)	11.9 ± 0.7	11.9 ± 0.8	12.0 ± 0.5	0.271

^a^ History of ipsilateral renal or ureteral calculi episodes.

The calculi were located in the upper, middle and lower ureter in 79, 16, and 79 patients (45.4%, 9.2%, and 45.4%), respectively. The total ESWL treatment success rate was 65.5%. Patients with older age (p = 0.003); larger stone length, stone width (p < 0.001), and stone burden (p < 0.001); radiopaque calculus (p = 0.001); history of ipsilateral calculus episodes (p = 0.002); and lower ESWL frequency (p < 0.001) tended to experience treatment failure in univariate analysis (Table 1).

Table 2 shows the multivariate analysis by logistic regression model using possible predictors in the univariate analysis and potential predictors identified in the literature, including BMI, waist circumference, buttock circumference, and energy.

**Table 2:** Multivariate analysis by logistic regression model using possible predictors in the univariate analysis and potential predictors identified in the literatures.

	Odds ratio	95% Confidence interval	P value
Age (mean, years)	1.042	1.007–1.078	0.020
Waist circumference	1.021	0.962–1.083	0.496
Hip circumference	1.039	0.972–1.112	0.262
BMI	0.934	0.808–1.080	0.359
Recurrence^a^	2.699	1.281–5.687	0.009
Stone burden	3.499	1.284–9.530	0.014
Radiopaque calculi	2.351	1.049–5.267	0.038
Distal ureter stone	0.613	0.287–1.313	0.208

^a^ History of ipsilateral renal or ureteral calculi episodes.

Age (odds ratio [OR], 1.042; 95% confidence interval [CI], 1.007–1.078), history of ipsilateral renal or ureteral calculi episodes (OR, 2.669; 95% CI, 1.281–5.687), stone burden (OR, 3.499; 95% CI, 1.284–9.530), and radiopaque stone (OR, 2.351; 95% CI, 1.049–5.267) were significant predictors of ESWL failure in all patients.

## Discussion

NCCT is an effective and efficient confirmative tool for acute renal colic. Potential predictors measured by NCCT for successful ESWL treatment, including SSD, stone density, stone size, and stone location, have been studied in the past decade [3–5, 12–14]. A large comparative effectiveness trial demonstrated that initial ultrasonography for suspected urolithiasis was not inferior to initial CT with regard to high-risk diagnoses with complications, serious adverse events, and hospitalizations, and is also associated with lower cumulative radiation exposure [15]. Recent studies have challenged the role of immediate ED CT scanning[16]. In practice, NCCT is not always performed even in outpatient clinic settings, except for those with suspected alternative diagnoses. The clinical application of results yielded by NCCT in the literatures is impractical. Thus, we used easily accessible parameters including personal history, patient characteristics, and calculi characteristics to identify predictors of successful treatment. We found that older age, history of ipsilateral renal or ureteral calculi episodes, larger stone burden, and radiopaque stones significantly predicted treatment failure. The definition of successful treatment and the timing of radiographic exams vary among the literatures. Some studies defined clinically insignificant residual fragments of less than 2 to 4 mm as successful treatment, because up to 80% of patients with stones <4 mm in size experience spontaneous passage [5, 17–19]. However, other studies, including ours, follow the more strict definition of being totally stone-free on radiography [14, 20–23]. The success rate in our study is 65.5%, which is comparable to those reported in the literature.

Several studies have reported that there is a tendency towards failure of ureter stone fragmentation in older patients, whereas some studies reported that age was not associated with treatment failure[19, 24]. In the present study, older patients were likely to experience treatment failure with a small increase in the OR (OR, 1.042; 95% CI, 1.007–1.078). The reason for this might be that aging ureters have different proportions of muscle, fibrous tissue, and elastic tissue, which may interfere with the passage of stone fragments [25]. We recorded whether the patients had experienced previous ipsilateral renal or ureteral calculi episodes, although the exact nature of calculi episodes was not clarified. Our results revealed that patients with previous ipsilateral calculus episodes had significantly higher treatment failure rates (OR, 2.669; 95% CI, 1.281–5.687). In the literature, long-term ureteral stone impaction and endoscopic procedures are associated with stricture formation [26]. Residual stone fragments might also be an etiological factor in stricture formation [27]. Strictures result from a mucosal inflammatory process involving tissue adherence or fibrosis following urothelial injury or urinary extravasation[26]. Some patients with ureteral strictures are asymptomatic [28]. It is implied that ESWL treatment failure in these patients might be related to potential ureteral stricture or ureteral structural changes. Thus, patients with a history of ipsilateral ureteral stone episodes warrant careful evaluation and clinical decision-making.

The effect of patients’ habitus has been studied. Studies have demonstrated that the BMI and SSD of patients are significant predictors for successful ESWL outcomes [3, 4]. Recently, Kang et al. reported an “optimal” SSD is a positive predictor [5]. Obesity might affect the efficacy of ESWL in different ways. For patients in the supine position, the ureters may move downwards significantly[21]. In addition to SSD and BMI, WC and BC were also studied. BC was also found to be a predictor of treatment failure [21]. In our study, we evaluated the patients’ habitus with BMI, WC, and BC. However, we could not find any significant differences in success rates among these variables.

Stone burden is a well-known predictor for ESWL treatment failure. This can be evaluated by measurement of maximal stone length, stone width, stone area, or stone volume on NCCT [21, 22, 24, 29]. The current study also showed that stone burden (OR, 3.499; 95% CI, 1.284–9.530) is one of the covariates in the multivariate regression and a significant predictor for ESWL failure.

Stone location has been considered as a possible predictor for successful treatment. Some studies showed better outcomes for stones in the proximal ureter than those in the middle or distal ureter[24]. The authors of these studies imputed the lower success rates for middle and distal ureteral stones to difficulties in focusing; stone location was not a predictor of treatment outcome in the current study.

In this study, we demonstrated the feasibility of ESWL without NCCT. Although the comparative values may vary among different geographical areas, a higher cost for NCCT (5,035 NTDs) than for plain radiography (200 NTDs) or IVU (1,430 NTDs) is obvious in the National Health Insurance data in Taiwan. The cost of ESWL is 26,920 NTDs. The mean medical cost of the treatment procedure in the present study was 32,413 NTDs. The total success rate in our study was 65.5%. The success rate of ESWL with IVU for radiolucent stones was 80%. IVU seemed ideal for intraoperative localization of the radiolucent stones and established a higher stone-free rate after the treatment. The lower medical cost of IVU than that of NCCT is also one of the advantages of adjunctive imaging modality.

The advantage of NCCT is that the stone density can be precisely evaluated in HU. It is believed that stone hardness is a significant predictor of treatment failure, although the cut-off value varies among the literature [23, 29]. We simply categorized the stones into radiolucent and radiopaque stones to represent stone density. We found that patients with radiopaque stones had significantly higher treatment failure rates (OR, 2.351; 95% CI, 1.049–5.267). The efficacy of ESWL for treating radiolucent ureteral calculi localized by intra-operative IVU has been studied, with reported outcomes comparable to those of radiopaque ureter calculi [6]. Previous studies demonstrated that radiolucency, defined by using plain radiography, may also present with a lower stone attenuation value on non-contrast CT [30]. The reported mean radiolucency of radiolucent urolithiasis was 358.25 ± 156 HU, which was much lower than those of cases with a poor chance of stone disintegration after ESWL reported in other studies [4]. This finding substantially supports our results and indicates that radiolucency is indeed a more cost-efficient predictive factor for successful treatment. In the present study, the patients did not undergo NCCT preoperatively; therefore, whether the stones are radiopaque or radiolucent is easier for physicians to determine, instead of density measured in HU. In accordance with our results, we suggest that ESWL could be considered a first-line treatment for symptomatic ureteral stones in patients with smaller radiolucent stones who have no history of ipsilateral renal or ureteral calculi.

## Conclusions

Age, history of ipsilateral renal or ureter calculi episodes, stone burden, and radiolucency were significant predictors for successful treatment of symptomatic ureteral stones with ESWL.

## Supporting information

S1 Table Original data of patients. (XLSX)
